# Potential for La Crosse virus segment reassortment in nature

**DOI:** 10.1186/1743-422X-5-164

**Published:** 2008-12-30

**Authors:** Sara M Reese, Bradley J Blitvich, Carol D Blair, Dave Geske, Barry J Beaty, William C Black

**Affiliations:** 1 Arthropod-borne and Infectious Diseases Laboratory, Department of Microbiology, Immunology and Pathology, Colorado State University, Fort Collins, Colorado, 80523-1692, USA; 2 Department of Veterinary Microbiology and Preventive Medicine, Iowa State University, Ames, IA, 50011-1250, USA; 3 Division of Vector-Borne Diseases, National Center for Infectious Disease Control and Prevention, Fort Collins, CO, 80522, USA; 4 La Crosse County Health Department, La Crosse, WI, 54601-3228, USA

## Abstract

The evolutionary success of La Crosse virus (LACV, family *Bunyaviridae*) is due to its ability to adapt to changing conditions through intramolecular genetic changes and segment reassortment. Vertical transmission of LACV in mosquitoes increases the potential for segment reassortment. Studies were conducted to determine if segment reassortment was occurring in naturally infected *Aedes triseriatus *from Wisconsin and Minnesota in 2000, 2004, 2006 and 2007. Mosquito eggs were collected from various sites in Wisconsin and Minnesota. They were reared in the laboratory and adults were tested for LACV antigen by immunofluorescence assay. RNA was isolated from the abdomen of infected mosquitoes and portions of the small (S), medium (M) and large (L) viral genome segments were amplified by RT-PCR and sequenced. Overall, the viral sequences from 40 infected mosquitoes and 5 virus isolates were analyzed. Phylogenetic and linkage disequilibrium analyses revealed that approximately 25% of infected mosquitoes and viruses contained reassorted genome segments, suggesting that LACV segment reassortment is frequent in nature.

## Background

In the 1970s, La Crosse virus (LACV family *Bunyaviridae*, genus *Orthobunyavirus*) emerged as a significant human pathogen in the upper Midwestern United States, and it is now the most common cause of pediatric arboviral encephalitis in the U.S [1]. LACV is maintained primarily in cycles between *Aedes triseriatus *and small mammals (usually chipmunks and tree squirrels). *Aedes triseriatus *develop a life-long infection, and infected females can transovarially transmit (TOT) the virus to their progeny [2,3]. TOT is perhaps the most important mechanism for maintenance and amplification of LACV in nature [4,5].

LACV has a tripartite, negative-sense RNA genome with the three segments designated large (L), medium (M), and small (S). The L segment encodes the RNA-dependent RNA polymerase [6], the M segment encodes a precursor polypeptide that is post-translationally cleaved to generate the G1 and G2 glycoproteins and the nonstructural protein NSm [7-10], and the S segment encodes the nucleocapsid protein and the small nonstructural protein NSs in overlapping reading frames [8].

LACV exhibits considerable evolutionary potential in nature. There are distinct geographic genotypes of the virus in different areas of the United States [11-14], and there is evidence that disease severity may be conditioned by certain LACV genotypes [13,15]. The evolutionary success of the LACV and other viruses in the family *Bunyaviridae *is attributed in part to their ability to adapt to varying conditions through genetic drift (intramolecular genetic changes) and genetic shift (segment reassortment).

Genetic drift occurs during genome replication and can result in viral diversity and altered fitness [16]. RNA virus replication yields multiple genetic variants, or quasispecies, which occur due to poor fidelity of the RNA polymerases and the lack of proofreading enzymes. The error-prone polymerase can provide an array of mutations, which allows constant adaptation to and selection by changes in the vector and vertebrate host.

Laboratory studies have demonstrated the occurrence of genetic shift (segment reassortment) in mosquitoes that have become dually infected by ingesting viruses of two different LACV genotypes, either simultaneously or within two days of each other [17]. LACV reassortant viruses can be isolated from up to 25% of dually infected *Ae. triseriatus *and the newly generated viruses can be transmitted. The potential for segment reassortment increases when a transovarially-infected mosquito takes a blood meal from a viremic host [18]. These mosquitoes can be orally super-infected, and can transmit the new reassortant viruses. The new reassortants might exhibit new characteristics such as altered host and vector ranges, new tropisms or virulence, and thus may be epidemiologically significant [5]. Segment reassortment is apparently restricted to closely related bunyaviruses, typically in the same serogroup [19-22].

Evidence has also been presented for reassortment between LACV genotypes in nature. For example, the genomes of 23 isolates of LACV were analyzed by oligonucleotide fingerprinting and categorized in terms of the degree of their RNA sequence relatedness [14]. One genotype (denoted type A) was isolated from mosquitoes from Wisconsin, Minnesota, Indiana, and Ohio and a second genotype (denoted type B) was isolated from mosquitoes from Minnesota, Wisconsin, and Illinois. A reassortant LACV isolated in Rochester, Minnesota contained the S segment of the B genotype, and the M and L segments of the A genotype.

Genome segment reassortment has also been demonstrated among other *Orthobunyaviruses *and in other *Bunyaviridae *genera. Ngari virus is a newly emerged reassortant virus associated with severe disease epidemics in Africa [23]. Sequence analysis of the three genomic RNA segments revealed that the S and L segments were derived from Bunyamwera virus, but the M segment was derived from Batai virus, an *Orthobunyavirus *that was first detected in Malaysia [24]. Group C *Orthobunyaviruses *also reassort [25]. Phylogenetic analysis revealed that Caraparu virus contains an S segment sequence that is nearly identical to that of the Oriboca virus and therefore is a natural reassortant virus. Reassortant Sin Nombre viruses (*Hantavirus*) have been detected in rodents in nature [26] and reassortant Crimean Congo hemorrhagic fever viruses (*Nairovirus*) have also been detected [27].

Although genome reassortment appears to occur frequently in the *Bunyaviridae *family, the epidemiologic consequences of these evolutionary events are poorly understood. In this study molecular epidemiological techniques were used to investigate the evolutionary and reassortment potential of LACV in field-infected mosquitoes from the upper Midwest of the United States.

## Results and discussion

### LACV infected mosquitoes and isolates analyzed

**Figure 1:**
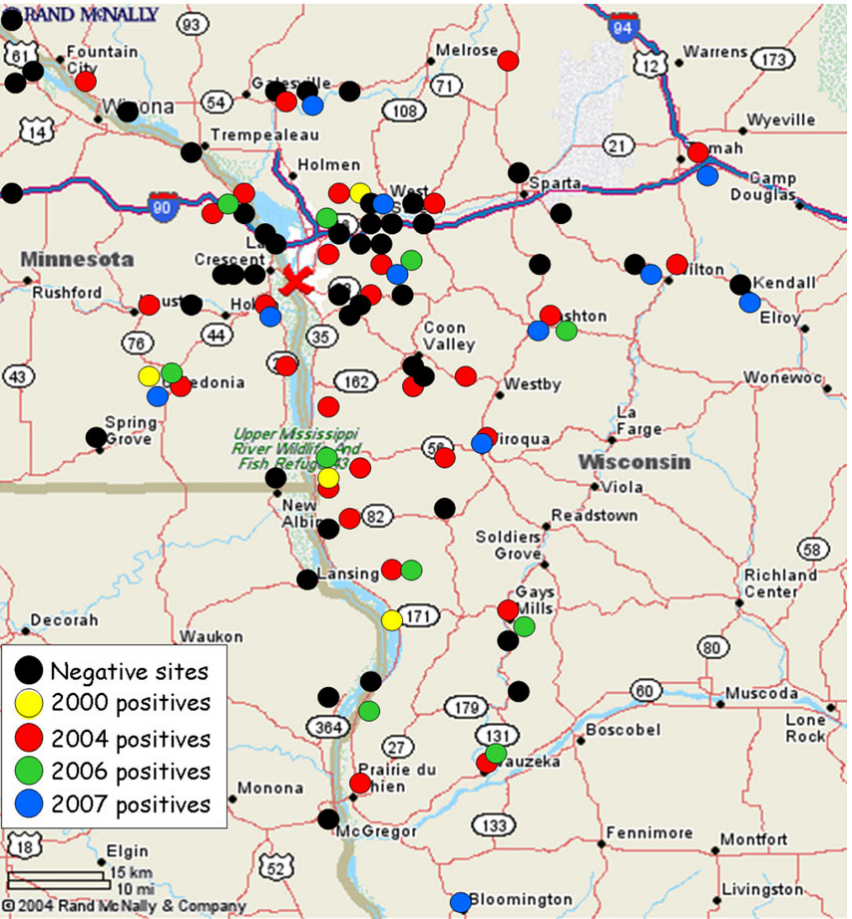
**Mosquito collection sites in Minnesota, Wisconsin, and Iowa**. Circles represent all collection sites. Yellow circles are the sites where LACV positive mosquitoes were collected in 2000, red circles are the sites where LACV positive mosquitoes were collected in 2004, green circles are the sites where LACV positive mosquitoes were collected in 2006, blue circles are the sites where LACV positive mosquitoes were collected in 2007 and black circles are the sites without positive mosquitoes. The "X" represents La Crosse, WI.

A total of 6,791 mosquitoes collected as eggs at 151 study sites in Wisconsin, Minnesota, and Iowa (Figure 1) were reared and tested for LACV antigen by immunofluorescence assay (IFA). Of these, 309 (4.6%) were positive. Viral RNA was amplified by RT-PCR from one to three mosquitoes from the selected sites listed in Table 1. Four LACV isolates from 1960, 1978, 2006 and 2007 were also examined in this study. The viruses from 2006 and 2007 were isolated from mosquitoes collected in the field. L, M, and S viral RNA (see Amplicon Cloning and Sequencing) was also amplified from the two virus isolates as well as directly from the two infected mosquitoes. The L, M, and S sequences from the viruses and the RNA amplified directly from the mosquitoes were identical (data not shown).

**Table 1:** *Ae. triseriatus *collection sites in Minnesota and Wisconsin of LACV-positive mosquitoes used in the analysis*

**Location/Site**	**County/State**	**Date Collected**	**Total Mosquitoes Collected**	**LAC+ Mosquitoes**	**%LAC+**
BC/Winona/2004	Winona, MN	6/18/2004	7	3	42.9

BEN/Lafayette/2007	Lafayette, WI	9/10/2007	50	3	6.1

BRS/Houston/2004	Houston, MN	6/29/2004	50	1	2.0

BWL/Houston/2004	Houston, MN	6/28/2004	38	2	5.3

CAL-B/Houston/2000	Houston, MN	5/1/2001	50	5	10.0

CAL-B/Houston/2004	Houston, MN	7/20/2004	50	2	4.0

CAL-D/Houston/2004	Houston, MN	7/20/2004	50	5	10.0

CAL-GA/Houston/2004	Houston, MN	7/20/2004	50	5	10.0

CAL-GA/Houston/2007	Houston, MN	8/27/2007	50	6	12.0

CAT/Monroe/2004	Monroe, WI	7/19/2004	50	1	2.0

DAK90/Winona/2004	Winona, MN	6/18/2004	38	3	7.9

ESO/Vernon/2004	Vernon, WI	7/22/2004	50	4	8.0

GAY120/Crawford/2004	Crawford, WI	7/22/2004	50	12	24.0

GRL/La Crosse/2004	La Crosse, WI	7/19/2004	50	1	2.0

HCS/Houston/2004	Houston, MN	8/2/2004	42	1	2.4

HHS/Houston/2004	Houston, MN	7/2/2004	50	1	2.0

H0/Vernon/2004	Vernon, WI	6/21/2004	24	1	4.2

INNB/La Crosse/2000	La Crosse, WI	5/1/2001	50	2	4.0

INNSL/La Crosse/2004	La Crosse, WI	6/28/2004	20	3	15.0

LAXCC/La Crosse/2004	La Crosse, WI	6/28/2004	30	2	6.7

LRHE/La Crosse/2000	La Crosse, WI	5/1/2001	50	2	4.0

MCBB/La Crosse/2004	La Crosse, WI	7/8/2004	50	2	4.0

MCP/La Crosse/2004	La Crosse, WI	6/17/2004	35	2	5.7

NAT/Crawford/2004	Crawford, WI	7/12/2004	50	3	6.0

NFCS/Crawford/2004	Crawford, WI	7/19/2004	50	1	2.0

OTS/La Crosse/2004	La Crosse, WI	7/19/2004	50	3	6.0

RRA/Houston/2004	Houston, MN	7/12/2004	50	3	6.0

RCS/Crawford/2004	Crawford, WI	6/21/2004	50	6	12.0

SHR/Vernon/2004	Vernon, WI	6/21/2004	50	1	2.0

SRS/La Crosse/2004	La Crosse, WI	7/19/2004	50	7	14.0

SST/La Crosse/2004	La Crosse, WI	7/19/2004	50	2	4.0

SVP/La Crosse/2004	La Crosse, WI	7/26/2004	50	4	8.0

SVP/La Crosse/2006	La Crosse, WI	8/31/2006	50	1	2.0

TFP/La Crosse/2004	La Crosse, WI	7/19/2004	41	17	41.5

VSA/La Crosse/2000	Vernon, WI	5/1/2001	50	1	2.0

VSB/La Crosse/2004	La Crosse, WI	6/21/2004	50	5	10.0

WKCS/Crawford/2004	Crawford, WI	6/21/2004	27	3	11.1

WSB/La Crosse/2004	La Crosse, WI	7/20/2004	47	2	4.3

WBF/Monroe/2004	Monroe, WI	7/19/2004	50	8	16.0

*Fifty mosquitoes were tested for LACV antigen from most sites. There were 11 sites with less than 50 adult mosquitoes.

### Rates and patterns of molecular evolution

**Figure 2:**
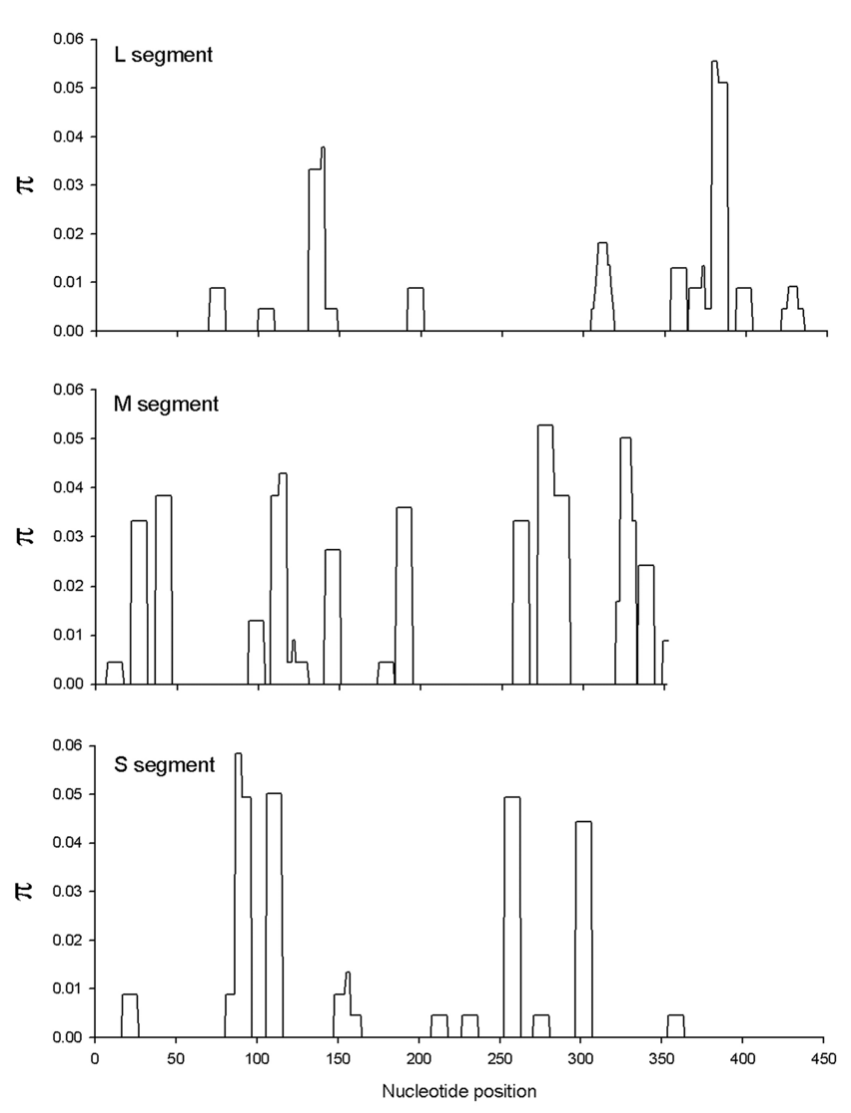
**Nucleotide diversity (π) of the LACV S, M and L segment sequences amplified from field-infected mosquitoes**.

The numbers of sequences analyzed and the number of segregating sites in each segment are shown in Table 2. The greatest nucleotide diversity (π) was seen in the M segment, twice that in the S segment and thrice that in the L segment. The distributions of these polymorphisms are shown in Figure 2. What is most noteworthy is that all three segments had more replacement than synonymous substitutions. In the L segment the diversity among replacement substitutions (π_a_) was actually 3.24 times larger than the diversity among synonymous substitutions (π_s_). The location and amino acid replacements are listed in Table 3. These trends suggest that some form of positive selection is operating on amino acid substitutions in all three segments.

**Table 2:** Polymorphisms and substitution rates in the L, M and S sequences amplified from field-infected mosquitoes

Segment analyzed	No. of sequences (this study)	No. of unique sequences	No. of segregating sites (syn.:rep.)	π ± std. dev	π_s _(potential synonymous sites)	π_a _(potential replacement sites)	π_a_/π_s_
**L segment**	45	12	19 (6:13)	0.00388 ± 0.00067	0.00141 (96.9)	0.00457 (350.1)	3.24
**M segment**	45	16	21 (7:14)	0.01154 ± 0.00102	0.01248 (77.25)	0.0113 (279.75)	0.90

**S segment**	45	9	13 (4:9)	0.00583 ± 0.00051	0.01091 (90.2)	0.00446(323.8)	0.41

**Table 3:** Nonsynonymous mutations found in sequences of LACV RNA that was RT-PCR amplified from field collected mosquitoes

**Segment**	**Genome location (nt)**	**Nucleotide Change**	**Amino Acid Change**
L	252	C → A	Pro → His

L	282	C → A	Pro → Glu

L	313	G → A	Met → Ile

L	321	T → C	Tyr → Ala

L	374	A → G	Thr → Ala

L	489	A → G	Asp → Gly

L	490	T → C	Arg → Gly

L	536	A → G	Asn → Asp

L	547	T → G	Phe → Cys

L	555	A → G	Lys → Arg

L	561	T → C	Ser → Leu

L	576	T → G	Phe → Cys

L	608	G → A	Ala → Thr

M	1663	A → G	Ile → Met

M	1749	G → A	Asn → Ser

M	1754	T → C	Tyr → His

M	1782	A → G	Asp → Gly

M	1815	T → C	Val → Ala

M	1826	T → C	Ser → Pro

M	1866	A → G	His → Arg

M	1881	T → C	Val → Ala

M	1887	A → G	Asn → Ser

M	1898	T → C	Cys → Arg

M	1913	T → C	Trp → Arg

M	1958	A → G	Thr → Ala

M	1961	A → G	Lys → Glu

M	1964	T → C	Phe → Leu

S	209	T → C	Phe → Ser

S	273	A → C	Lys → Asn

S	298	A → G	Ile → Val

S	340	A → G	Asn → Asp

S	347	A → G	Asp → Gly

S	400	T → C	Tyr → His

S	419	A → T	Glu → Val

S	445	A → G	Thr → Ala

S	463	G → A	Ala → Thr

The program Tipdate [28] estimated the molecular evolutionary rate (substitutions/site), the absolute molecular evolution rate (substitutions/site/year) of each segment and the age of the dataset (the time in years since the sequences evolved from a common ancestral sequence)(Table 4). The absolute evolution rate was most rapid in the S segment, 480 times greater than the rate in the L segment and 4.8 times greater than the rate in the M segment. Both the M and S segments appear to be of similar ages, while the L segment appears to predate both by ~400,000 years.

**Table 4:** Evolution rates in the L, M and S sequences of LACV.

Segment Analyzed	Molecular evolution rate (substitutions/site)	Absolute molecular evolution rate (substitutions/site/year)	Age of tree (years)
**L segment**	6.7 × 10^-6^	1.0 × 10^-5^	421,842
**M segment**	1.11 × 10^-4^	9.93 × 10^-4^	25,108
**S segment**	3.95 × 10^-5^	4.8 × 10^-3^	28,003

### Haplotype determination

**Figure 3:**
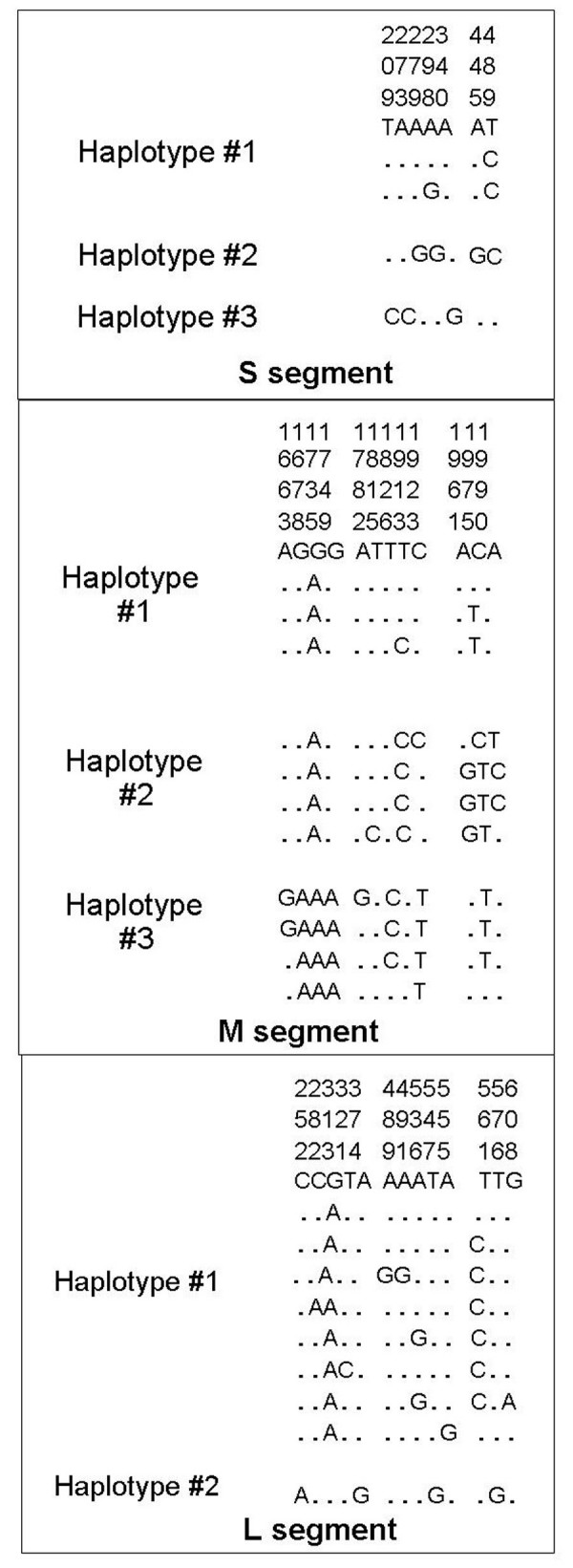
**LACV S, M, and L segment haplotype determination**. Phylogenetic analyses yielded three haplotypes for the S segment, three haplotypes for the M segment, and two haplotypes for the L segment. The genome position is provided above the genetic sequence.

The haplotype grouping system was determined through a conservative phylogenetic analysis. The system identified three S haplotypes based on seven polymorphic sites, five of which were nonsynonymous mutations. The three haplotypes identified in the M segment were based on twelve polymorphic sites, seven of which were nonsynonymous. For the L segment, two haplotypes were identified based on thirteen polymorphic sites, twelve of which were nonsynonymous substitutions (Figure 3).

### Phylogenetic analysis

**Figure 4:**
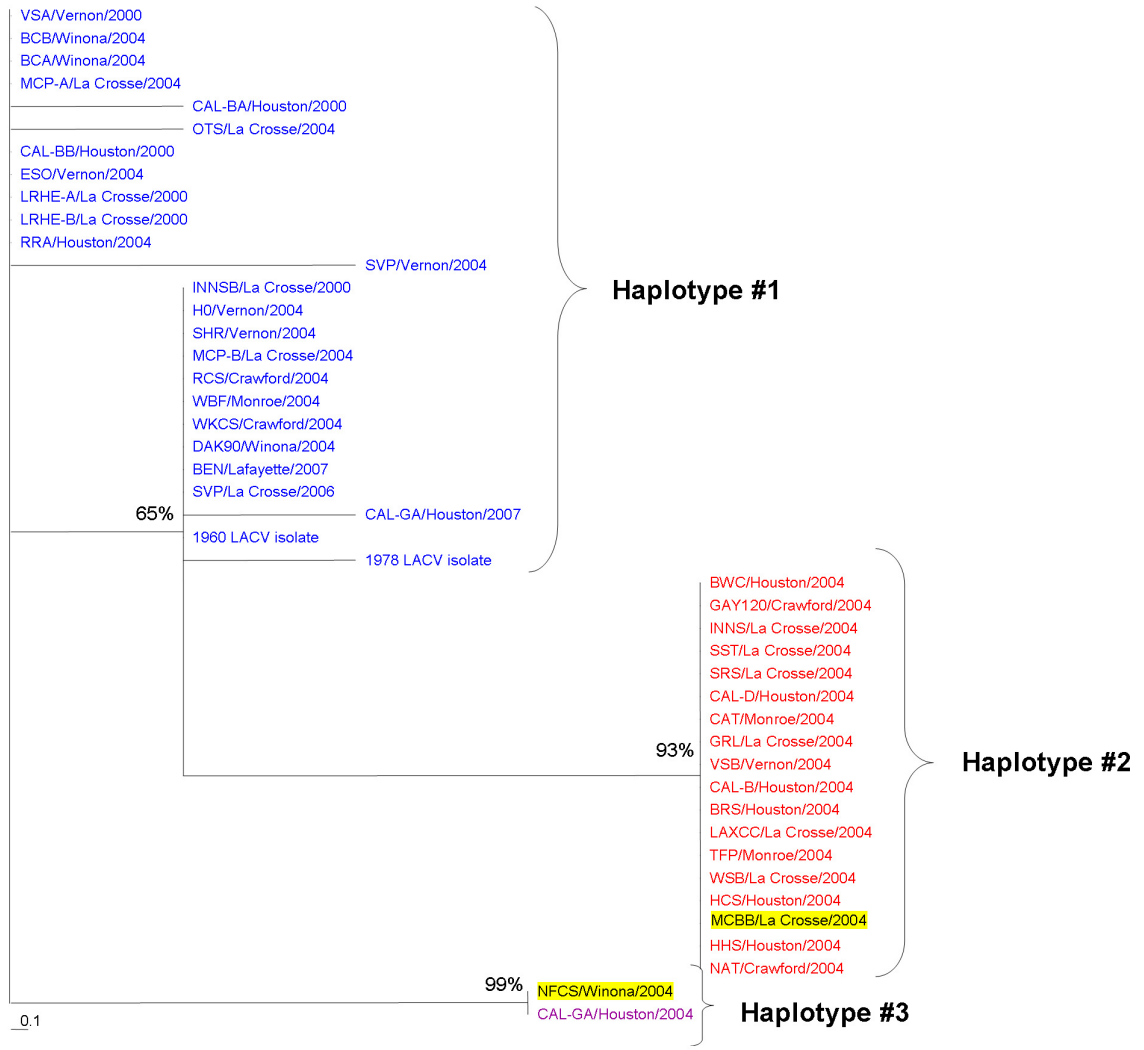
**S segment (nucleotides 190–604) phylogenetic tree**. Maximum parsimony phylogenetic analysis of LACV RNA amplified from field collected mosquitoes from 2000 and 2004 and from LACV isolates from 1960, 1978, 2006, and 2007. Bootstrap values were assigned for 100 replicates represented by the numbers on the branches. Colors represent haplotypes determined for the S segment and are continued for the M and L segments. The two highlighted samples are examples of segment reassortment.

**Figure 5:**
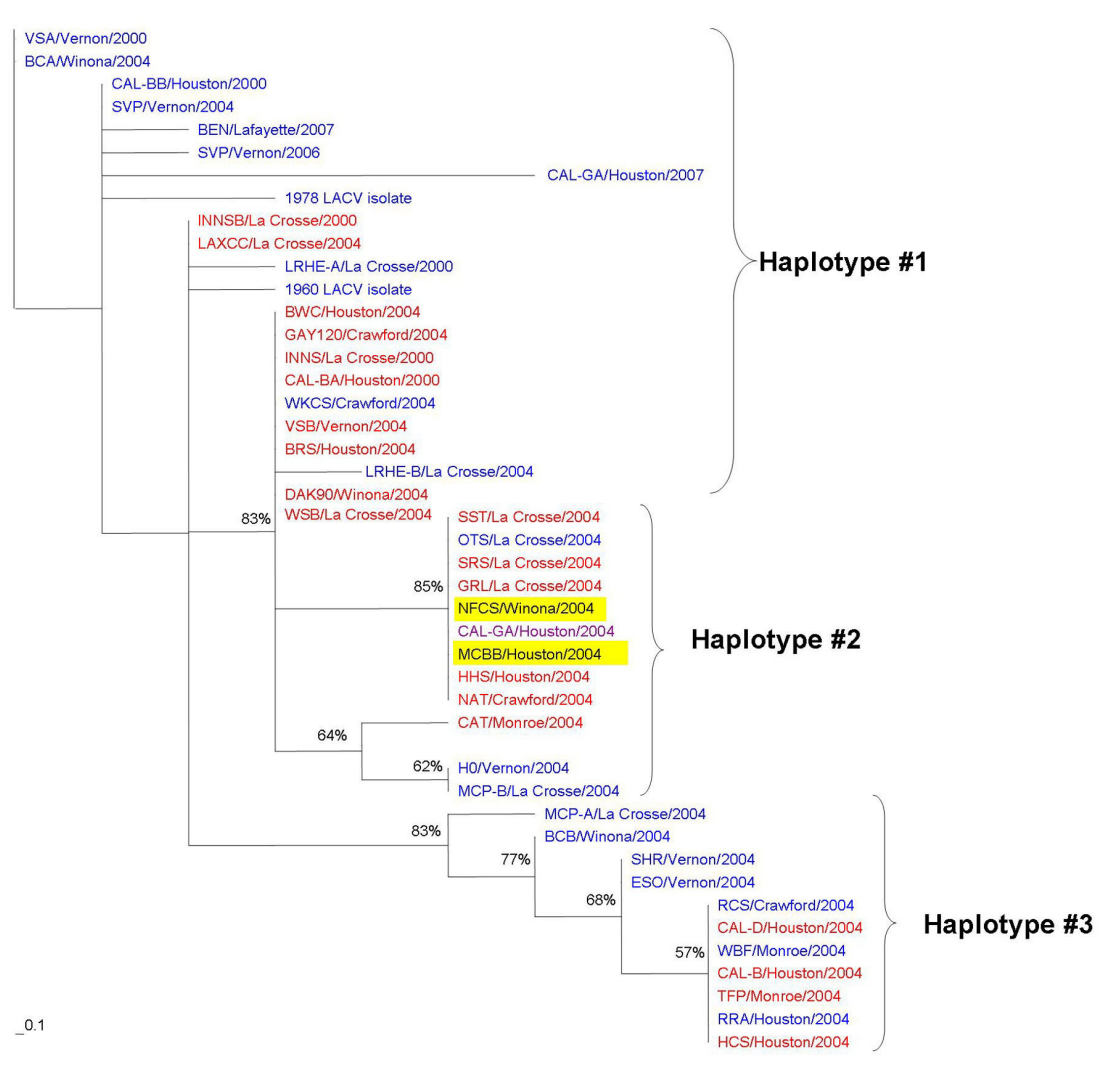
**M segment (nucleotides 1637–1994) phylogenetic tree**. Maximum parsimony phylogenetic analysis of LACV RNA amplified from field collected mosquitoes from 2000 and 2004 and from LACV isolates from 1960, 1978, 2006, and 2007. Bootstrap values were assigned for 100 replicates represented by the numbers on the branches. Colors represent haplotypes determined for the S segment and are continued for the M and L segments. The two highlighted samples are examples of segment reassortment.

**Figure 6:**
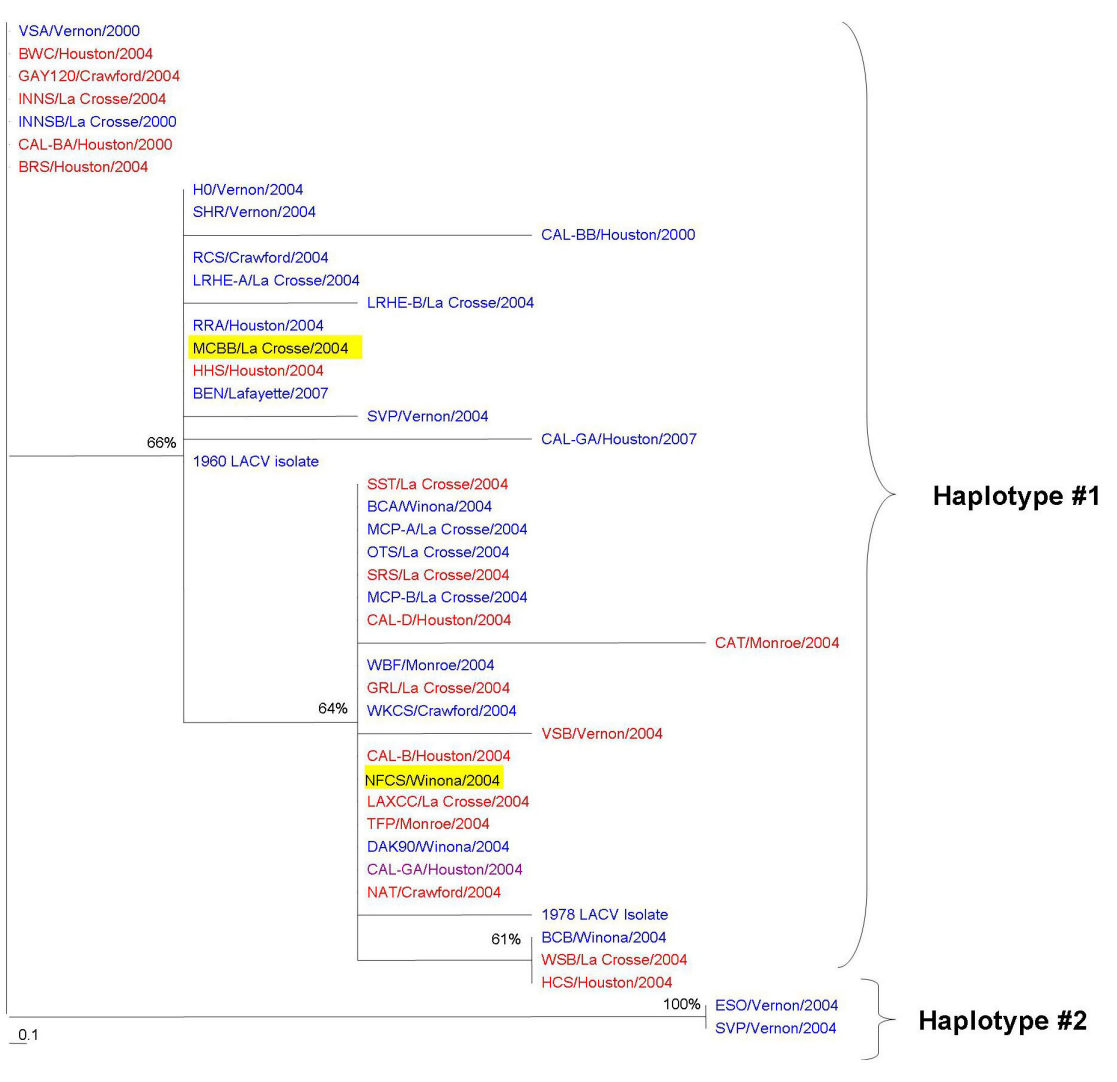
**L segment (nucleotides 179–625) phylogenetic tree**. Maximum parsimony phylogenetic analysis of LACV RNA amplified from field collected mosquitoes from 2000 and 2004 and from LACV isolates from 1960, 1978, 2006, and 2007. Bootstrap values were assigned for 100 replicates represented by the numbers on the branches. Colors represent haplotypes determined for the S segment and are continued for the M and L segments. The two highlighted samples are examples of segment reassortment.

Maximum parsimony phylogenetic trees were established using amplified sequences from each of the three segments. Comparison of the clades on the three maximum parsimony trees provides evidence for the potential for transmission of reassortant viruses by the infected *Ae. triseriatus *(Figures 4, 5, 6). If there were no reassortants, the three genome segments from each infected mosquito would have appeared in the same clade. A number of mosquitoes contained viral genome segments that clustered into different clades in each of the trees. For example, the S segment from the sample MCBB/La Crosse/2004 was in haplotype #2 (red), the M segment in haplotype #2 (predominantly red) and the L segment in haplotype #1 (mixture of red and blue). Another example is the LACV RNA from the mosquito collected in NFCS/Winona/2004. The S segment was in haplotype #3 (purple), the M segment in haplotype #2 (predominantly red), and the L segment in haplotype #1 (mixture of red and blue). These suggest that segment reassortment had occurred. The distribution of the sequences in the phylogenetic trees for all three segments would be identical if reassortment had not occurred; however, the phylogenetic trees are highly variable when the S, M and L segment tree topologies are compared.

### Linkage disequilibrium analysis

**Figure 7:**
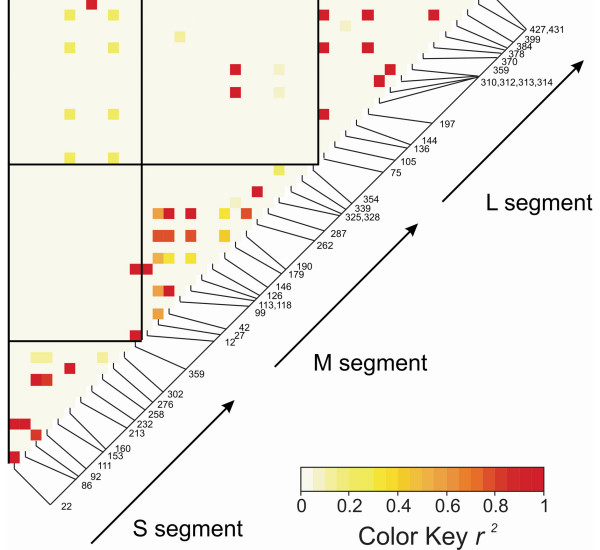
**A heat map of linkage disequilibrium within and among the LACV S, M, and L segments**. The matrix is read according to the nucleotide position of segregating sites displayed along the diagonal. Low disequilibrium coefficients are represented by white or yellow and high disequilibrium coefficients are represented by orange or red.

A linkage disequilibrium analysis was performed within and among the S, M, and L segments. Figure 7 is a heat diagram in which low disequilibrium coefficients are represented by light yellow squares and high disequilibrium coefficients are represented by red squares. The matrix is read according to the nucleotide position of segregating sites displayed along the diagonal. For example in Figure 7, the lowest square connects sites S22 (segregating site 22 from the S segment) and S86 and it is red. This corresponds to an r^2 ^of 1.00 and these sites are in complete linkage disequilibrium. In contrast, squares linking site S359 with all other sites are light yellow indicating that all sites in S are in equilibrium with S359. The triangles along the diagonal in Figure 7 contain many red squares indicating that many sites within a segment are in disequilibrium. Thus our coverage of each of the segments appears adequate.

The squares in Figure 7 indicate patterns of disequilibrium among segments. In contrast to the large amounts of disequilibrium found within each of the segments, there is very little disequilibrium among segments. Between S and M there are 192 (12 S sites × 16 M sites) possible interactions but only two of these are in disequilibrium: S359 with M12 and M126. Otherwise 99% of possible interaction between S and M are in equilibrium indicating extensive reassortment between these segments. Between S and L there are again 192 possible interactions but only two in disequilibrium; these are S232 and L427. All other possible interaction between S and L are in equilibrium indicating reassortment between these segments. Between M and L there are again 256 possible interactions but only two of these are in disequilibrium: M179 with L312 and L314. All other possible interaction between M and L are in equilibrium indicating reassortment between these segments.

An independent heterogeneity χ^2 ^analysis (Table 5) was performed to test this pattern. There were 3 S clades, 3 M clades and 2 L clades; thus there were 18 possible segment combinations corresponding to each row in Table 5. The observed column is the number of times that a segment combination occurred in the 45 samples. Eight of the combinations were in disequilibrium but 10 were in equilibrium (in bold) supporting an inference of frequent reassortment. In total, eleven of the 45 (24.4%) samples were in linkage equilibrium

**Table 5:** LACV segment reassortment occurs in field collected mosquitoes as revealed by a linkage disequilibrium analysis*

**S**	**M**	**L**	**Obs**	**p-value**
1	1	1	14	***

**1**	**1**	**2**	**1**	

1	2	1	3	***

1	2	2	0	

**1**	**3**	**1**	**6**	

1	3	2	1	*

2	1	1	7	***

2	1	2	0	

2	2	1	7	***

2	2	2	0	

**2**	**3**	**1**	**4**	

2	3	2	0	

3	1	1	0	***

3	1	2	0	

3	2	1	2	***

3	2	2	0	

3	3	1	0	***

3	3	2	0	

			45	

* p-value ≤ 0.01, ** p-value ≤ 0.001, *** p-value ≤ 0.0001

### LACV segment reassortment in nature

Both phylogenetic and linkage disequilibrium analyses revealed that LACV RNA genome segments had undergone reassortment in 24% of mosquitoes and isolates analyzed. This is remarkable and illustrates the exceptional evolutionary potential and genetic diversity of *Bunyaviridae *viruses in nature. One possible reason for this could be the ability of *Ae. triseriatus *to become dually infected. When mosquitoes ingest two different LACV isolates simultaneously or sequentially within four hours, 100% become dually infected [17]. Even at 48 hours post-initial bloodmeal, 27% of mosquitoes that ingest a second virus become dually infected before a barrier to superinfection develops. In addition, when transovarially-infected mosquitoes ingested a bloodmeal containing a heterologous LACV, 19% became dually infected [18]. These experiments suggest that dual infection can occur frequently through both oral and transovarial infection and therefore increase the possibility of segment reassortment in vectors. The newly evolved viruses are also efficiently transmitted [17]. These experiments were performed in a controlled laboratory setting, but they demonstrate the potential for segment reassortment to occur frequently in nature.

Although the analyses demonstrate the potential for reassortment, most of the sequences used were from RNA amplified directly from the infected mosquitoes and not from virus isolates. The reassortment frequency detected in this study could have resulted from analysis of RNA quasispecies sequences in the mosquito. However the L, M, and S sequences obtained from the virus isolates as well as those directly amplified from the infected mosquitoes in 2006 and 2007 were identical. This suggests that 1) the genome sequence obtained by direct amplification of the viral RNA from the mosquito is the dominant viral sequence in the mosquito as well as in infectious virus and 2) that the estimation of reassortment frequency was not confounded by potential RNA quasispecies in the mosquitoes. Estimating the frequency of reassortment of LACV in nature would be improved by analysis of plaque-purified viruses isolated from the mosquitoes, preferably from their saliva or ovaries, which are the epidemiologically significant organs of transmission.

In this regard, we were unsuccessful in isolating LACV from most field mosquitoes. The reasons for this are unknown; however, there are several potential explanations for this. Eggs were collected in the field and stored in a hot warehouse for variable periods of time awaiting shipment to Colorado. As soon as the eggs reached AIDL, they were placed in the insectary, hatched and reared. Environmental factors in the collection and shipping process could contribute to loss of virus titer. An additional complication could have been the isolation method. In previous studies, virus was isolated by inoculation of samples into suckling mouse brains. Cell culture assays are likely not as sensitive. Low virus titer, titer loss during processing, and insensitive isolation methods, likely contributed to the inability to isolate virus from mosquitoes.

## Conclusion

There are important public health implications of reassortment in LACV-infected *Ae. triseriatus *in the field. LACV reassortants could be more virulent and could have altered vector species and vertebrate host ranges. New viruses could create new arbovirus cycles with potentially significant epidemiological consequences [5]. For example, the geographic distribution of LACV is currently determined by the distribution of *Ae. triseriatus *and chipmunks and tree squirrels. If a new virus established a transmission cycle that involved a mosquito species that fed more aggressively on humans, increased human infections could occur. If a new reassortant virus was more virulent or exhibited different tissue tropisms, infections could become clinically significant in both adults and children. For example, a new reassortant virus could replicate more efficiently in humans, resulting in greater viremia titers and more efficient infection of the central nervous system. Determination of the evolutionary potential of LACV through genetic shift may permit prediction of the epidemiologic consequences of these events.

These studies illustrate the significant evolutionary and epidemic potential of viruses in the family Bunyaviridae. Viruses in this family have contributed inordinately to the list of newly emerged viruses [29], and they will likely continue to do so in the future.

## Methods

### Egg collection

*Aedes triseriatus *eggs were collected from five oviposition traps in each of 151 sites in Minnesota (n = 37), Wisconsin (n = 108) and Iowa (n = 6). Sites were established in areas where LACV encephalitis cases occurred or areas that contained clusters of people judged by the La Crosse County Public Health Department to be at risk for infection (e.g. wooded areas adjacent to houses with children, schools, or playgrounds). Mosquito eggs that had entered diapause in fall 2000 were collected in the spring of 2001. Mosquito eggs were also collected between mid-June and August of 2004, 2006 and 2007. Eggs were collected in Crawford, La Crosse, Monroe, Vernon, Lafayette and Iowa counties in Wisconsin; Winona, Houston, and Grant counties in Minnesota; and Clayton and Allamakee counties in Iowa (Figure 1). Eggs were transported to the insectaries at the Arthropod-borne and Infectious Diseases Laboratory (AIDL) at Colorado State University (CSU); Fort Collins, CO. Eggs were hatched immediately and reared to adults.

### Immunofluorescence assay (IFA)

To determine if mosquitoes were infected, mosquito heads were severed, squashed onto acid-washed microscope slides, and fixed in acetone. Heads were assayed for LACV antigen by direct IFA using LACV-specific polyclonal antiserum [30].

### LACV-positive mosquitoes

Viral RNA from 40 mosquitoes was analyzed, including 34 field collected mosquitoes from 2004 and six field collected mosquitoes from 2000.

### LACV strains

Previously isolated LACV strains were also used in the analysis. The 1960 LACV isolate was isolated originally from the brain of a child who died from LACV encephalitis in La Crosse, WI and it was passed five times in suckling mouse brains (SMB). A 1978 LACV (78V-8853) was isolated from an *Ae. triseriatus *mosquito from Rochester, MN and passed once in Vero cells and twice in SMB. LACV was isolated from mosquitoes collected in the field in WI and MN in 2006 and 2007.

### LACV isolation

The LACV-positive mosquitoes were triturated with a pellet pestle (Fisher Scientific) in a 1.5 ml microcentrifuge tube containing 1 ml of minimum essential medium (MEM) (Gibco), 2% fetal bovine serum, 200 μg/ml penicillin/streptomycin, 200 μg/ml fungicide, 7.1 mM sodium bicarbonate, and 1× nonessential amino acids. The homogenate was centrifuged for 10 minutes at 500 × g to form a pellet.

Cell monolayers of Vero cells were grown in six-well plates at 37°C in an atmosphere of 5% CO_2_. Supernatant from the centrifuged mosquito homogenate (0.2 ml) was added to one well in a six-well plate, incubated at 37°C for one hour. Following the incubation, 5 ml of medium were added to each well.

### Plaque purification

The virus isolates from 2006 and 2007 were plaque purified using monolayers of Vero cells in six-well plates [31]. Virus isolates were serially diluted 10^-1 ^to 10^-6 ^and 200 μl of each virus dilution was added to individual wells and incubated at 37°C for 1 hour. The virus inoculum was removed and 5 ml of overlay was added to the well. After six days of incubation at 37°C in 5% CO_2_, 200 μl of the detection solution, methylthiazolyldiphenyl-tetrazolium bromide (MTT) (5 mg/ml in PBS), was added to each well. The plates were incubated overnight and visible plaques were picked and placed in 1 ml of MEM with 0.2% FBS for 1 hr at 37°C. An aliquot of the medium from the wells was added to Vero cells, and the presence of virus confirmed by detection of cytopathic effect.

### RNA purification from mosquitoes

The posterior half of each mosquito abdomen was individually homogenized in 500 μl of Trizol (Invitrogen, Carlsbad, CA), using a pellet pestle (Fisher Scientific, Pittsburg, PA), and then total RNA was extracted according to manufacturer's instructions.

### RNA purification from virus isolates

The medium and cells from wells with plaque purified virus were removed and placed in a 15 ml conical tube and centrifuged at 3000 rpm for 10 minutes. The supernatant was removed and the cell pellet was resuspended in 500 μl of Trizol (Invitrogen, Carlsbad, CA). Total RNA was extracted according to manufacturer's instructions.

RNA from the 1960 and 1978 LACV isolates was prepared by infection of C6/36 cell cultures at a multiplicity of infection of 0.01. Three days post-infection, cells were scraped into the medium, centrifuged and cell pellets were resuspended in 500 μl of Trizol for RNA extraction.

### Amplification by reverse transcription-PCR

Portions of the LACV S, M, and L RNA segments were transcribed to cDNA using Superscript II reverse transcriptase (Invitrogen, Carlsbad, CA) and amplified by PCR using Ex *Taq *DNA polymerase (Takara, Shiga, Japan) according to manufacturer's instructions. The primers specific for the S segment (forward: 5'-GCAAATGGATTTGA TCCTGATGCAG-3', reverse: 5'-CTTAAGGCCTTCTTCAGG TATTGAG-3') amplified a 462 nucleotide region (nucleotides 144 to 604) of the nucleocapsid and NSs genes. This region was selected because it was the most variable region of the published S sequences. The S segment is 984 nucleotides in length, so the amplified region encompasses almost half the entire segment. The primers specific for the M segment (forward: 5'-CCAAAAGCAACAAAAGAAAGA-3', reverse: 5'- CTGAAGGCATGAT GCAAAG-3') amplified a highly variable 411 nucleotide region in the 5' half of the G1 gene (nucleotides 1585 to 1995) [32]. The primers specific for the L segment (forward: 5'-GCATGTGTAGCCAAGGATATCGATG-3', reverse: 5'-CAGTCTTGCACCAGG GTGCTGTAAG-3') amplified a 487 nucleotide region (nucleotides 140 to 626). These primers also were selected to amplify the most variable region of the L segment. Primers specific for the *Ae. triseriatus *ribosomal protein RpL34 mRNA were used as a positive control. PCR was performed as follows: 94°C for 5 minutes, 35 cycles of 94°C for 1 minute, 55°C for 1 minute and 72°C for 1 minute followed by a final extension at 72°C for 8 minutes.

### Amplicon cloning and sequencing

PCR products were separated by electrophoresis in 1% agarose gels with TAE buffer, visualized with ethidium bromide, excised and extracted using the Powerprep Express Gel Extraction kit (Marligen Biosciences, Ijamsville, MD) according to manufacturer's instructions. PCR products were inserted into the pCR4-TOPO cloning vector (Invitrogen, Carlsbad, CA) and resulting plasmids were used to transform competent TOP10 *E. coli *cells (Invitrogen, Carlsbad, CA). Cells were grown on LB agar containing ampicillin (50 μg/ml) and kanamycin (50 μg/ml). Colonies were screened for inserts by PCR amplification using the original primers and positive products were purified using QIAquick spin columns (Qiagen, Valencia, CA). Three to five cDNA clones per segment from each mosquito were sequenced in both directions using the ABI PRISM dye terminator cycle sequencing kit (Applied Biosystems, Foster City, CA) and the ABI 310 DNA automated sequencer at Macromolecular Resources, CSU. A 415 nucleotide region of the S segment (nucleotides 190–604), a 358 nucleotide region of the M segment (nucleotides 1637–1994), and a 447 nucleotide region of the L segment (nucleotides 179–625) were sequenced.

### Haplotype determination

Genetic haplotypes were established for each of the three segments through maximum parsimony analysis, sequence identity matrix, neighbor joining distance matrix, and ratio of synonymous to nonsynonymous substitutions.

### Statistical analyses

#### 1. DNA polymorphism and nucleotide substitution rates

For each segment, the computer program DnaSP 4.5 [33] estimated π the average number of nucleotide differences among all pairwise comparisons of sequences [34], equation 10.5]. π was also estimated separately for synonymous (π_s_) and replacement substitutions (π_s_). The rate of molecular evolution (substitutions/site/year) was estimated using the program TipDate [28]. Tipdate analyzes sequences of RNA viruses that have been obtained at different dates to provide a maximum likelihood estimate of the absolute rate of molecular evolution. The program assumes a molecular clock to estimate the date of the most common ancestor.

#### 2. Linkage Disequilibrium Analysis

Linkage disequilibrium is a measure of the degree to which substitutions in a segment occur independently of one another. Substitutions that occur together in a segment at a rate predicted by their independent frequencies are in linkage equilibrium. Substitutions that occur more or less often than expected by random chance are considered to be in linkage disequilibrium. Linkage disequilibrium also tests whether sampling a portion of a genome segment is representative of the whole segment. Linkage equilibrium is detected when different parts of a segment are evolving independently and sequencing a portion of the segment may not provide a representative sample of the whole.

A linkage disequilibrium analysis was also performed to determine if entire segments assort randomly, thereby suggesting segment reassortment. Segment are in disequilibrium when some combinations of segments occur together in a mosquito more or less often than would be predicted by their independent frequencies. The first step is to determine the number of times segment, S_*i*_, Mj, and L_*k *_appear in the same mosquito, where for example, S_*i *_is a unique sequence of the segment.

(1) T_*ijk *_= the number of times haplotype *i, j*, and *k *occur in a mosquito.

E_*ijk *_is the number of times haplotype *i*, *j*, and *k *are expected to occur in a mosquito.

(2) E_*ijk *_= N (p_*i *_p_*j *_p_*k*_),

where p_*i *_is the frequency of S_*i *_in the mosquito population and N is the number of mosquitoes. Linkage disequilibrium (D_*ijk*_) was then estimated.

(3) D_*ij *_= (N/N-1)*(T_*ijk *_- E_*ijk*_)/N

A Hill and Robertson correlation coefficient R_*ijk *_was determined [35].

(4) R_*ijk *_= D_*ij*_/((p_*i*_(1-p_*i*_))(p_*j*_(1-p_*j*_))(p_*k*_(1-p_*k*_))^1/2 ^

The squared correlation coefficient (R_*ijk*_^2^) was used as a metric of disequilibrium because it ranges from zero (linkage equilibrium) to one (linkage disequilibrium). Linkage disequilibrium patterns among all polymorphic sites were plotted on a heat map using the LDheatmap program in R [36]. A chi-square statistic (χ^2^_Link_) and the corresponding level of significance were calculated for each combination of haplotypes to test the hypothesis that the individual haplotype combinations are in linkage equilibrium.

(5) χ^2^_Link(1d.f) _= (N R_*ijk*_^2^)

#### 3. Maximum Parsimony analysis

Maximum parsimony phylogenetic analysis was performed using the Phylogenetic Analysis Using Parsimony (PAUP) 4.0b10 package [37]. The phylogenetic trees indicate the branches that appeared in the majority of the 100 bootstrap pseudo replications and the frequency with which these appear among replications. A maximum parsimony phylogenetic tree was created for each of the three genome segments.

## Competing interests

The authors declare that they have no competing interests.

## Authors' contributions

SR and BrB carried out virological and molecular characterization of viruses. SR and WB conducted phylogenetic analyses and statistical analyses. DG identified field collection sites and managed field collections and logistics. CB, BaB, and WB conceived, designed, and managed the study. All authors participated in the preparation of the MS and approve its submission to Virology Journal.
